# An 8-gene qRT-PCR-based gene expression score that has prognostic value in early breast cancer

**DOI:** 10.1186/1471-2407-10-336

**Published:** 2010-06-28

**Authors:** Iker Sánchez-Navarro, Angelo Gámez-Pozo, Álvaro Pinto, David Hardisson, Rosario Madero, Rocío López, Belén San José, Pilar Zamora, Andrés Redondo, Jaime Feliu, Paloma Cejas, Manuel González Barón, Juan Ángel Fresno Vara, Enrique Espinosa

**Affiliations:** 1 Unidad de Investigación, Instituto de Investigación Sanitaria IdIPAZ, Hospital Universitario La Paz (Paseo de la Castellana, 261), Madrid (28046), Spain; 2 Service of Oncology, IdIPAZ, Hospital Universitario La Paz (Paseo de la Castellana, 261), Madrid (28046), Spain; 3 Service of Pathology, IdIPAZ, Hospital Universitario La Paz (Paseo de la Castellana, 261), Madrid (28046), Spain; 4 Service of Statistics, IdIPAZ, Hospital Universitario La Paz (Paseo de la Castellana, 261), Madrid (28046), Spain; 5 Chair of Oncology & Palliative care, Facultad de Medicina, Universidad Autónoma de Madrid (Arzobispo Morcillo n° 2 y 4), Madrid (28029), Spain

## Abstract

**Background:**

Gene expression profiling may improve prognostic accuracy in patients with early breast cancer. Our objective was to demonstrate that it is possible to develop a simple molecular signature to predict distant relapse.

**Methods:**

We included 153 patients with stage I-II hormonal receptor-positive breast cancer. RNA was isolated from formalin-fixed paraffin-embedded samples and qRT-PCR amplification of 83 genes was performed with gene expression assays. The genes we analyzed were those included in the 70-Gene Signature, the Recurrence Score and the Two-Gene Index. The association among gene expression, clinical variables and distant metastasis-free survival was analyzed using Cox regression models.

**Results:**

An 8-gene prognostic score was defined. Distant metastasis-free survival at 5 years was 97% for patients defined as low-risk by the prognostic score versus 60% for patients defined as high-risk. The 8-gene score remained a significant factor in multivariate analysis and its performance was similar to that of two validated gene profiles: the 70-Gene Signature and the Recurrence Score. The validity of the signature was verified in independent cohorts obtained from the GEO database.

**Conclusions:**

This study identifies a simple gene expression score that complements histopathological prognostic factors in breast cancer, and can be determined in paraffin-embedded samples.

## Background

A key aspect of the management of women with early breast cancer is the selection of adjuvant therapy, which is guided by the use of prognostic factors included in the guidelines[1,2]. However, the use of these criteria leads to unnecessary treatment in many women. Gene expression profiles may improve prognostic and predictive information in breast cancer patients, so that adjuvant chemotherapy is given only to those with the higher risk. A number of such profiles are currently available and two of them, the 70-Gene Signature (MammaPrint™) and the Recurrence Score (OncoType™) are being evaluated in phase III studies [3,4].

The major drawbacks to the widespread use of gene expression profiling include reservations regarding their cost/effectiveness ratio and their lack of widespread availability. For some profiles, the need for fresh-frozen biological samples adds to these drawbacks. We have previously demonstrated that 1) quantitative reverse-transcriptase polymerase chain reaction (qRT-PCR) can be used to assess the genes included in the 70-Gene Signature and 2) commercially available probes can be used on formalin-fixed, paraffin-embedded (FFPE) samples to determine the 70-Gene Signature, the Recurrence Score and the Two-Gene Ratio [5,6].

In the present study, we sought to identify a gene expression profile based on the genes included in the 70-Gene Signature, the Recurrence Score and the Two-Gene Ratio. Our hypotheses were as follows: 1) commercially available assays can be used to quantify gene expression by qRT-PCR using RNA extracted from FFPE tissues and 2) it is possible to find a prognostic gene expression profile using a reduced number of genes.

## Methods

### Patients and clinical data

Case selection was performed retrospectively. To be included in the study, patients had to have stage I or II (TNM classification, 2002) estrogen and/or progesterone receptor-positive invasive ductal breast cancer. Patients also had to have tissue samples available for gene expression analysis, and were required to have received appropriate therapy (according to standard protocols) during the inclusion period. Appropriate therapy was defined as either mastectomy or tumorectomy plus adjuvant radiotherapy, as well as adjuvant hormonal therapy for 5 years in all patients and adjuvant anthracycline-based chemotherapy in N+ or in N0 patients with poor prognostic features. A minimum follow-up of 5 years was also required for all patients who did not relapse. Institutional approval from our ethics committee was obtained before the study was initiated.

### RNA isolation and cDNA synthesis

The biological specimens used in this study were FFPE samples stored at the Pathology Department of our institution. An experienced pathologist evaluated H&E preparations to select samples containing at least 70% tumor cells. Fifteen 5-μm sections from each FFPE sample were de-paraffinized with xylene and washed with ethanol. RNA was then extracted with the Master Pure™ Kit (Epicentre). We normalized to total RNA input; therefore, first-strand cDNA was synthesized from 1 μg of total RNA using random primers, according to the High Capacity cDNA Reverse Transcription Kit protocol (Applied Biosystems).

### Quantitative RT-PCR

qRT-PCR amplifications were performed with TaqMan Gene Expression Assay products in an ABI PRISM 7900 HT Sequence Detection System (Applied Biosystems). The reactions were carried out using TaqMan Low Density Arrays (TLDAs, Applied Biosystems). TLDAs have proved appropriate for assessment of FFPE samples [7]. For quality control, samples with insufficient tumor tissue, insufficient RNA (less than 1 μg), or a weak RT-PCR signal (average cycle threshold for the reference genes greater than 35) were excluded. A preliminary analysis was performed to compare gene expression measurements between paired FF and FFPE samples. The mean coefficient of variation for the same assays performed on different days or in different batches was less than 3%.

### Gene selection

We configured a TLDA series to analyze the genes included in the 70-Gene Signature[8], the Recurrence Score [3] and the Two-Gene Ratio[9] with available TaqMan Gene Expression Assays (Supplementary table 1).

**Table 1:** Clinical characteristics of the included patients*

Median age: 58 years, range 29-82	
	**Number of patients (%)**

T	
1	77 (50.3%)
2	76 (49.7%)

N	
0	96 (62.7%)
1	57 (37.3%)

Stage	
I	61 (39.9%)
IIa	51 (33.3%)
IIb	41 (26.8%)

Hormone receptor status	
ER+/PgR-	30 (19.6%)
ER+/PgR+	110 (71.9%)
ER+/PgR unknown	12 (7.9%)
ER-/PgR+	1 (0.7%)

Grade	
1	29 (18.9%)
2	64 (41.8%)
3	59 (38.6%)
Unknown	1 (0.7%)

Chemotherapy	
No chemotherapy	56 (36.6%)
CMF	42 (27.4%)
Anthracycline-based	55 (35.9%)

* All patients received adjuvant tamoxifen.

CMF: cyclophosphamide, methotrexate, 5-fluorouracil

### Calculation of gene expression

Average cycling threshold (Ct) values[10], were obtained using SDS 2.2 software (Applied Biosystems). The maximum Ct value was set at 40. Ct values were normalized using four housekeeping genes (IPO8, HMBS, POLR2A and SDHA). The relative expression level of each target gene was expressed as ΔCt = Ct_ref _- Ct_gene_[11]. Reference-normalized expression measurements were adjusted by defining the lowest expression value as 0, with subsequent 1-unit increases reflecting an approximate doubling of the RNA [3].

### Methodology used to find a reduced profile

First, genes displaying poor correlation in gene expression levels between fresh frozen and FFPE samples were discarded, as described elsewhere [12]. We computed a statistical significance level for each gene based on a univariate proportional hazards model [13] with the aim of identifying genes whose expression was significantly related to the distant metastasis-free survival (DMFS). Genes related to DMFS were subsequently filtered based on their p-values (p < 0.005) and the correlation between them. We then selected genes with the lowest p-values in each correlation group [3]). These selected genes were used to develop a gene expression-based prediction model of recurrence risk using the supervised principal component method of E. Bair and R. Tibshirani [14]. Leave-one-out cross-validation was used to evaluate the predictive accuracy of the profile. It was also used for initial screening of the genes. The cutoff point was established prior to gene selection including all samples. We assessed cutoff points leaving from 10% to 90% of patients in the low risk group and increasing by 10% each time. To test the statistical significance of each cutoff point, the p-value of the log-rank test statistic for the risk groups was evaluated using 1000 random permutations. Analyses were performed in BRB-ArrayTools v3.6.1 developed by R. Simon and A. Peng. We used the REMARK guidelines to ensure that the manuscript provided relevant information [15]. Additional description of the methods can be found in supplemental PDF file (Additional file 1, Additional methods).

### Statistical analyses

To evaluate the prognostic value of the reduced gene score in our patient population, survival curves were estimated using Kaplan-Meier analysis and compared using the log-rank test. Univariate and multivariate Cox proportional-hazards analyses were also employed to create a final model that included the tumor grade (1 vs. 2 and 1 vs. 3), size (≤2 cm vs. >2 cm) and nodal status (0 vs. 1-3 positive nodes). Like other studies examining gene profiles in breast cancer, DMFS was the primary end point.

Two-way contingency-table analyses, as well as calculations of Cramer's V statistic, were also performed to measure the strength of the association between the 8-Gene Score (the score we identified) and the 70-Gene Signature and the Recurrence Score[16]. To assess model accuracy at five years, Harrell's bias-corrected concordance index was calculated. Models were refit 500 times using the bootstrap resampling technique. The concordance index is the percentage of patient pairs in which the predicted and observed outcomes coincide, such that c = 0.5 represents agreement by chance and c = 1.0 represents perfect discrimination[17]. Concordance is identical to the area under a receiver operating characteristic curve (ROC)[18].

To define the continuous relationship between the 8-gene Score and the 5-year DMFS rate, Gray's piecewise-constant time-varying coefficients model was used[19]. A linear tail-restricted cubic spline function was generated using "R" v 2.4.1 with the Design software package v2.0-12.

### Independent data sets

Four independent databases that are available online were used as validation sets: 1) NKI [8], downloaded from the Rosetta Inpharmatics Web page http://www.rii.com/publications/2002/nejm.html, 2) SWE (GSE1456)[20], 3) UPP (GSE4922)[21], and 4) LOI (GSE6532) [22], downloaded from the NCBI GEO data repository http://www.ncbi.nlm.nih.gov/projects/geo/index.cgi. To apply our qRT-PCR reduced gene score to these microarray data sets, the expression values of each set were z-score transformed [23]. Expression values were adjusted, with the lowest expression value defined as 0 and other values scaled accordingly. Per-gene normalization within the validation cohorts was performed using median values obtained in the discovery cohort [24]. Survival curves were then estimated.

SPSS v9.1 software package and "R" v 2.4.1 (with the Design software package v2.0-12) were used for all statistical analyses. All *P *values were two-sided, and *P *< 0.05 was considered statistically significant.

## Results

We considered 736 patients for inclusion in the study and a total of 153 patients were finally included. Additional file 2 Table S1 includes raw data of gene expression, whereas Additional file 3 Table S2 checks the REMARK recommendations along the manuscript. Table 1 describes the clinical features of and therapies received by the 153 patients. The median age was 58 years and the median follow-up was 91 months. Thirty-four patients (22%) had a distant relapse, of whom 17 died and 7 were lost to follow-up after the relapse. Among 119 patients who did not have a distant relapse, four had a local/regional recurrence that was successfully treated with surgery.

We first selected 53 genes that exhibited highly correlated expression levels in FF and FFPE samples. Seventeen genes were subsequently filtered based on their *P*-values related to DMFS. A model was built using these 17 genes (data not shown). Models with 10, 8, 7 or even fewer genes also demonstrated a good separation between groups at a low and high risk of distant recurrence. We selected a score based on 8 genes because it demonstrated the best performance. The 8-gene Score was calculated for each sample using reference-normalized expression measurements based on the following equation:

8-gene Score = 0.1936*DTL + 0.2176*ECT2 + 0.0454*MTDH + 0.1329*PRC1 + 0.0556*RFC4 - 0.1913*SCUBE2 - 0.0443*STK32B - 0.1182*ZNF533.

Table 2 displays the names and coefficients of these 8 genes. Increased expression levels of DTL, ECT2, MTDH, PRC1 and RFC4 were associated with shorter disease-free survival, whereas increased expression levels of SCUBE2, STK32B, and ZNF533 were associated with longer disease-free survival. All of these genes are included in the 70-Gene Signature, although SCUBE2 is also included in the Recurrence Score.

**Table 2:** Identification of the genes included in the 8-Gene Score

Gene	p-value	Score coefficient	RefSeq	Assay ID
DTL	1.2e-06	0.1936	NM_016448.2	Hs00212788_m1

ECT2	2.8e-06	0.2176	NM_018098.4	Hs00216455_m1

MTDH	2.9e-06	0.0454	NM_178812.2	Hs00757841_m1

PRC1	<1e-07	0.1329	NM_003981.2	Hs00187740_m1

RFC4	0.0002592	0.0556	NM_181573.2	Hs00427469_m1

SCUBE2	0.0005634	-0.1913	NM_020974.1	Hs00221277_m1

STK32B	0.0004406	-0.0443	NM_018401.1	Hs00179683_m1

ZNF533	2.13e-05	-0.1182	NM_152520.4	Hs00332216_m1

BRB ArrayTools was used to define a cutoff point for risk stratification. We assessed cut-off points leaving from 10% to 90% of patients in the low risk group increasing by 10% each time. Best performance was obtained when 60% of the patients were allocated to the low-risk group. Patients with a total score <2.86 were assigned to the low risk of recurrence group and patients with a score ≥2.86 constituted the high risk of recurrence group. The *P*-value of the log-rank test used to calculate recurrence risk between risk groups based on 1000 permutations was 0.018.

**Figure 1:**
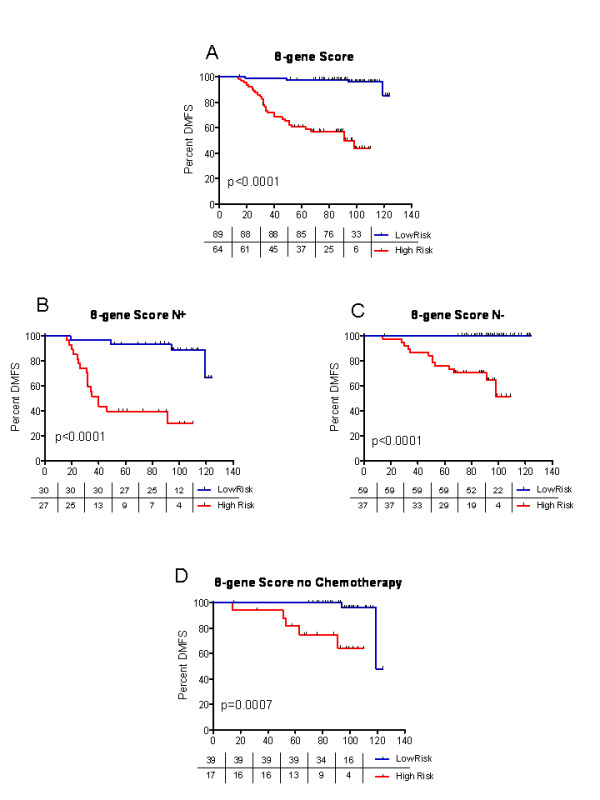
**Kaplan-Meier curves showing distant metastasis-free survival (DMFS) for low-risk and high-risk patient groups (as defined by the 8-gene Score)**. A: all 153 patients included in this study B: patients with positive lymph nodes C: patients with negative lymph nodes D: patients who did not receive chemotherapy

**Figure 2:**
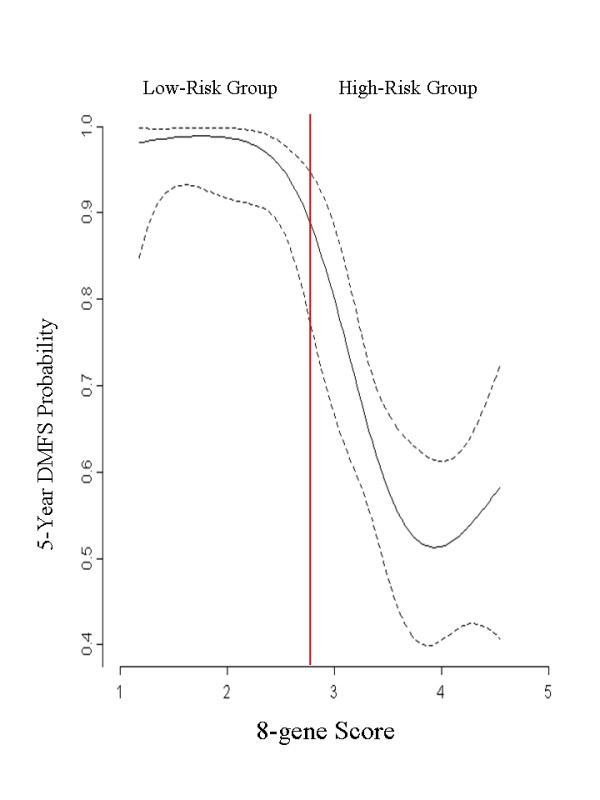
**Distant metastasis-free survival (DMFS) rates as a continuous function of the 8-gene Score**. The red line indicates the cutoff point. Dashed lines indicate the 95% confidence interval.

DMFS at five years was 97.7% for patients in the low-risk group and 60.6% for patients in the high-risk group (HR: 20.4, CI 95%: 6.2 - 67.5; p < 0.001) (Figure 1A). DMFS at five years decreased continuously as the 8-Gene Score increased (Figure 2). Overall survival at five years was also calculated, and was found to be 98.9% for the low-risk group and 86.6% for the high-risk group (HR: 7.496, CI 95%: 2.4 - 23.4; p < 0.001). A univariate analysis evaluating the effects of pathological factors on DMFS and OS is presented in Additional file 4 Table S3.

A sub-analysis was performed to evaluate the influence of lymph node status on DMFS (Figures 1B and 1C). In patients with lymph node involvement, the 8-gene Score included half of each group (low- vs. high-risk), and DMFS at five years was 93.3% vs. 39.5%, respectively. In women without lymph node involvement, the five-year DMFS rates were 100% vs. 75.7%, respectively. We also evaluated the performance in the population of patients receiving just endocrine therapy and no chemotherapy (figure 1D).

The multivariate Cox proportional hazards analysis included the 8-gene Score, tumor size, nodal status and tumor grade. The 8-gene Score was able to predict DMFS (Table 3), indicating that this gene expression profile added important prognostic information beyond that provided by clinical factors. Lymph node status remained the only clinical factor with significant independent predictive value.

**Table 3:** Multivariate analysis of factors associated with distant metastasis-free survival in the 8-gene Score, the 70-Gene Signature and the Recurrence Score

	8-Gene Score		
	
	HR	CI 95%	p value
Tumor size	1.31	0.52 - 3.3	0.55

Nodal status	2,87	1.38 - 6.01	**0.005**

Grade (2 vs.1) (3 vs. 1)	0.60 1.42	0.11 - 3.16 0.28 - 7.15	0.227 0.555 0.665

8-gene Score	15.61	4.41 - 55.17	**<0.001**

	**70-Gene Signature**		
	
	HR	CI 95%	p value

Tumor size	1.4	0.57 - 3.42	0.46

Nodal status	2.70	1.30 - 5.62	**0.008**

Grade (2 vs.1) (3 vs. 1)	0.82 2.349	0.160 - 4.181 0.473 - 11.672	0.113 0.810 0.297

70-Gene Signature	3.505	1.33 - 9.23	**0.011**

	**Recurrence Score**		
	
	HR	CI 95%	p value

Tumor size *	1.17	0.48 - 2.82	0.72

Nodal status **	2.49	1.20 - 5.16	**0.014**

Grade (2 vs1) (3 vs 1)	0.68 2.26	0.13 - 3.45 0.49 - 10.40	**0.043** 0.644 0.292

Recurrence Score (Interm. vs. Low risk) (High vs. Low risk)	4.46 8.18	0.78 - 25.33 1.76 - 37.86	**0.021** 0.091 **0.007**

*Tumor size: >2 cm vs. ≤2 cm. **Nodal status: positive (1-3) vs negative.

We used Cramer's V statistic to asses the concordance between other gene profiles [3,4] and the 8-gene Score. The correlation between the 8-gene Score and the 70-Gene Signature was 0.65 and the correlation was 0.58 for the Recurrence Score and 0.30 for the Two-Gene Index. These results indicate that there is a strong correlation between the 8-gene Score and both the 70-Gene Signature and the Recurrence Score. To assess the discriminative capability of each prognostic profile at five years, Harrell's bias-corrected concordance index was calculated. The calculated values were as follows: 8-gene Score = 0.81, Recurrence Score = 0.73, 70-Gene Signature = 0.70, and Two-Gene Index = 0.59.

**Figure 3:**
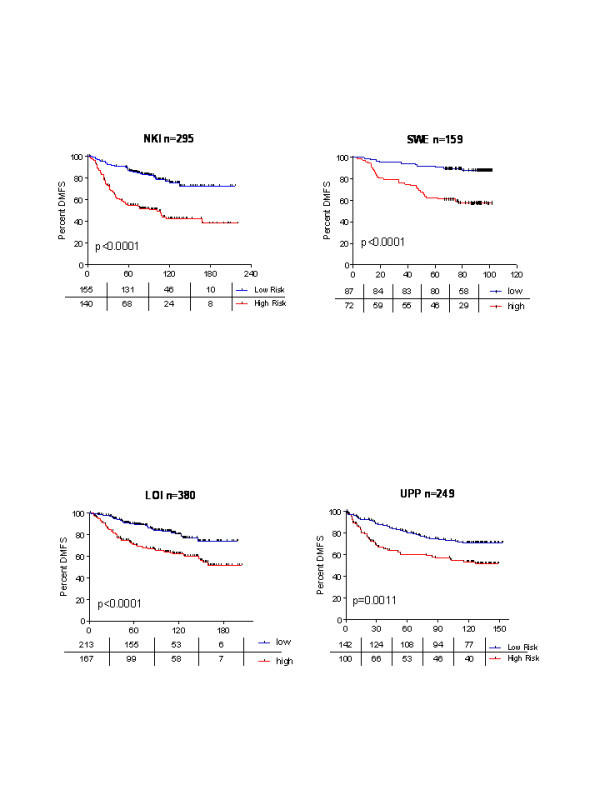
**Kaplan-Meier curves showing distant metastasis-free survival (DMFS) for patients included in four databases available online**.

We then applied the 8-gene Score to an online database from the Dutch Cancer Institute (NKI) that has been previously used to compare several gene profiles[16]. The 8-gene Score identified significant differences in DMFS for the entire group of 295 patients (Figure 3), as well as for the N+, N-, and ER + subgroups based on their score (Additional file 5 table S4). In comparison with the 70-Gene Signature, the 8-gene Score categorized more patients as being low-risk, and DMFS was a bit lower for all groups. However, if the cutoff value was modified to include the same number of patients in the low-risk category as in the 70-Gene Signature, then the results for DMFS were virtually identical.

We subsequently analyzed the performance of the 8-gene Score in three additional data sets: SWE (GSE1456)[20], UPP (GSE4922)[21], and LOI (GSE6532)[22]. Although the use of other external databases does not constitute a formal validation, it may provide insight about the performance of the gene set. A similar approach has recently been reported in bladder carcinoma [25]. In all data sets, the 8-Gene Score was able to identify significant differences in DMFS (figure 3). In all cases, the 8-gene Score assigned more than 55% of the patients to the low-risk group. It predicted a DMFS rate of >90% in the LOI and SWE data sets, and >80% in the UPP data set.

## Discussion

In the present study, we propose a qRT-PCR-derived prognostic score based on the expression levels of eight genes (the 8-Gene Score) for use in women with early breast cancer. The score can be determined using FFPE samples. It identified patients at high risk of recurrence in our series of patients as well as in four independent databases available online.

In comparison with women included in the other online databases, all of our patients received tamoxifen and two thirds were also treated with chemotherapy. The fact that the 8-gene Score worked well in these databases reflects its strength as a prognostic tool that can be used in large populations. Our gene expression profile was developed using hormonal receptor-positive tumors and, although it was derived from a signature validated in both positive and negative tumors [4], it should not be used for women with hormone receptor-negative tumors.

The genes included in the 8-gene Score are also part of the 70-Gene Signature, but the former should not be considered a scaled down version of the latter, because the 8-Gene Score is applicable to FFPE samples and can be obtained using commercially available probes. Models with fewer than 8 genes performed similarly to the 8-gene Score, but the 8-gene Score was finally selected because it was more stable than smaller models and, at the same time, did not contain redundant information.

DTL, ECT2, MTDH, PRC1 and RFC4 have all been previously implicated in breast cancer; SCUBE2 and STK32B deletions have been found to be related to mental deficits in humans; and ZNF533 has been found to be associated with the Hedgehog signaling pathway in Zebrafish [26-35]. Of note, MTDH activation by 8q22 genomic gain promotes chemoresistance and metastasis of poor-prognosis breast cancer [36]. The eight genes themselves could play an important role in the prognosis of breast cancer. Alternatively, their expression levels could vary in response to changes in the expression of more influential genes. This means that rather than identifying the ultimate genetic cause of a cancer, gene expression profiles may instead provide information about the molecular consequences of critical mutations. In our population, the 8-gene Score identified groups of patients whose DMFS rates differed, both in the whole patient population and in the node positive and node negative subgroups (figure 1). A sub-analysis of patients receiving endocrine therapy and no chemotherapy showed the possible relevance of the 8-gene profile for decision of chemotherapy.

According to the results from the NKI series [16], the 8-gene Score performed as well as the 70-Gene Signature (Additional file 5 table S4). The DMFS and OS rate values were higher using the 70-Gene Signature, probably due to the different number of patients assigned to low- versus high-risk groups in each profile, as well as to the fact that many women in the NKI series did not receive adjuvant therapy [37].

The 8-gene Score did not perform as well in other data sets as it did in ours, probably because of substantial differences in the use of adjuvant therapy. For instance, many women with estrogen receptor-positive tumors did not receive adjuvant hormonal therapy in the UPP series, and most did not even receive chemotherapy although over one third of these patients had positive lymph nodes[21]. The series by Loi et al. included women with hormone receptor-positive tumors who were treated with tamoxifen, but chemotherapy was not used in any patient [22]. Our profile was generated in a population of women who had a very good prognosis and received optimal adjuvant therapy, and therefore the cutoff values identified using this cohort may be a bit too optimistic for "sub-optimally" treated populations. This suggests that the 8-gene Score may provide not only prognostic but also predictive information. However, because our profile works as a continuous variable, the cutoff value can be changed to become less restrictive depending on the patient group to which it is applied.

Our study has some limitations. Whereas the 70-Gene Signature and the Recurrence Score have undergone extensive investigation and clinical use, the 8-gene Score is still undergoing initial development. Our retrospective series included a limited number of patients and the treatment they received was not uniform. Furthermore, without an independent validation series, we cannot rule out overestimation of the prognostic value of the 8-gene Score. The application of our score to external data sets may temper this limitation, but it is not a formal validation. Without such validation, the score cannot facilitate treatment choices, as it neither distinguishes between therapy benefit nor identifies a group of patients that do not need any therapy. The use of different populations in the discovery phase may certainly yield different profiles, but our point was to demonstrate that a small subset of informative genes could provide a significantly prognostic score.

The 8-gene Score correlated very well with two currently available gene profiles: the 70-Gene Signature and the Recurrence Score. The discrimination capacity (Harrell's C index) was high for all three profiles. The Two-Gene Index did not perform similarly to the 8-Gene Score in our cohort of patients, but this does not demonstrate inferiority. On the other hand, the performance of this index has recently been improved with the incorporation of five additional genes related to the tumor grade [38].

We feel that the 8-gene Score has three main advantages. First, it can be used in FFPE samples with commercial probes. Second, considering the modular nature of this platform, it would be easy and inexpensive to add further genes that might provide additional information, as has been done with the Two-Gene Index [38]. Next generation profiles could include both prognostic and predictive information, for instance. Finally, qRT-PCR using TLDAs can be easily implemented in the clinical setting.

## Conclusions

We have described an 8-gene prognostic Score that can be used in FFPE samples from patients with early hormone receptor-positive breast cancer. The main interest of this study is that it opens the door to the use of gene expression profiling based on small sets of genes in local laboratories.

## Competing interests and funding

The 8-gene Score is under patent OEPM P-200803509 (December 2008), "Method for the sub-classification of tumours".

This work was supported by FIS Grant PI050668 (Instituto de Salud Carlos III, Ministerio de Sanidad y Consumo, Spain) (EE, ISN, JAFV), Fundación SEOM grant 2006 (EE), Red Temática de Investigación Cooperativa en Cáncer (RTICC, RD06-0020-1022), FEDER funds and unrestricted grants from Astra-Zeneca, Bristol-Myers Squibb, Cephalon-Pharma, Glaxo SmithKline, Roche Farma, Sanofi-Aventis and Schering-Plough.

JAFV is supported by FIS Grant CP05/00248 from Fondo de Investigación Sanitaria (Instituto de Salud Carlos III, Ministerio de Sanidad y Consumo, Spain).

AGP is the recipient of a fellowship from the Ministerio de Educación y Ciencia, Spain. ISN is supported by Fundación para la Investigación del Hospital Universitario La Paz.

## Authors' contributions

ISN, AGP and JAFV helped designing the study, carried out the molecular studies and drafted the manuscript. RM and BSJ performed the statistical analysis. DH performed the pathological workup. AP, PZ, AR, JF, PC and MGB contributed to the design of the study. RL contributed to the laboratory workup. EE conceived and designed the study the study and drafted the manuscript. All authors read and approved the final manuscript.

## Pre-publication history

The pre-publication history for this paper can be accessed here:

http://www.biomedcentral.com/1471-2407/10/336/prepub

## Supplementary Material

Additional file 1 **additional methods**. further description of methods used to determine cutoff point and to find a reduced gene profile. Click here for file

Additional file 2 **Table S1, Raw data**. Table with list of genes included in the study, raw data of gene expression and clinical data Click here for file

Additional file 3 **Table S2, REMARK criteria**. checking of REMARK criteria for marker development throughout text Click here for file

Additional file 4 **Table S3, univariate analysis**. table displaying univariate analysis for distant metastasis-free survival and overall survival Click here for file

Additional file 5 **Table S4, DMFS in online databases**. table displaying percentages of patients in low and high risk groups, as well as distant metastasis-free survival percentages in online available databases. Click here for file
