# Supplementary material for: An 8-gene qRT-PCR-based gene expression score that has prognostic value in early breast cancer
Source: BMC Cancer. 2010 Jun 28;10:336. doi: 10.1186/1471-2407-10-336 (PMC2906483; doi:10.1186/1471-2407-10-336)
Supplement: Additional file 1 — additional methods. further description of methods used to determine cutoff point and to find a reduced gene profile. [file 1471-2407-10-336-S1.DOC]

Supplementary Methods

Regarding the cut-off point selection, it was optimized before gene selection including all samples. The whole process was validated using LOOCV by means of BRB Array Tools version 3.6, developed by Richard Simon & BRB-ArrayTools Development Team. The following text, extracted from BRB Array Tools Manual, version 3.6, shows the methodology we used:

The Survival Analysis Tool for finding genes whose expression is correlated with survival time fits proportional hazards models relating survival to each gene, one gene at a time and computes the p value for each gene for testing the hypothesis that survival time is independent of the expression level for that gene. Gene lists are created based on these p values in the same way as in the Class Comparison tools. The p values can be used to construct gene lists using multivariate permutation tests for controlling the number or proportion of false discoveries. Or the gene list can simply consist of the genes with p values less than a specified threshold (0.001 is default). For more information regarding the multivariate permutation tests for controlling the number or proportion of false discoveries, please see the preceding section on Multivariate Permutation Tests for Controlling Number and Proportion of False Discoveries.

The Survival Analysis Prediction Tool develops a gene expression based predictor of survival risk group. The number of risk groups and the risk percentiles for defining the groups are specified by the user. The survival risk groups are constructed using the supervised principal component method of E Bair and R Tibshirani (2004):

E Bair & R Tibshirani, Semi-supervised methods to predict patient survival from gene expression data, PLOS Biology 2:511-522, 2004).

This method uses a Cox proportional hazards model to relate survival time to k “super-gene” expression levels, where k is selectable by the user (usually 1-3). The “supergene” expression levels are the first k principal component linear combinations of expression levels of the subset of genes that are univariately correlated with survival. The user specifies the threshold significance level (e.g. 0.001) for selecting the genes to be used in computing the principal components. The significance of each gene is measured based on a univariate Cox proportional hazards regression of survival time versus the log expression level for the gene. After selecting the genes, the principal components are computed, and the k-variable Cox proportional hazard regression analysis is performed. This provides a regression coefficient (weight) for each principal component.

Having developed a supervised principal component model as described above, to compute a prognostic index for a patient whose expression profile is described by a vector x of log expression levels, the following steps are performed. First the components of the vector x corresponding to the genes that were selected for use in computing the principal components are identified. Then the k principal components are computed. These are just linear combinations of the components of x, with the weights of each linear combination having been determined from the principal component analysis described above. Finally, the weighted average of these k principal component values is computed, using as weights the regression coefficients derived from the k-variable Cox regression described above. That computation provides a prognostic index for a patient with a log expression profile given by a vector x. A high value of the prognostic index corresponds to a high value of hazard of death, and consequently a relatively poor predicted survival.

In order to evaluate the predictive value of the method, Leave-One-Out-Cross-Validation is used. A single case is omitted and the entire procedure described above is performed to create a prognostic index. This function is created from scratch on the training set with the one case removed, including determining the genes to be included in the calculation of the principal components. Having determined a prognostic index function for that training set, it is used to compute a prognostic index for the omitted observation. That value is compared to the prognostic index for the n-1 cases included in that training set (assuming that there are n distinct cases available in total). The prognostic index for the omitted patient is ranked relative to the prognostic index for the patients included in the cross-validated training set. The omitted patient is placed into a risk group based on his/her percentile ranking, the number of risk groups specified, and the cut-off percentiles specified for defining the risk groups. This analysis is repeated from scratch n times, leaving out a different case each time.

Having completed the computations described in the previous paragraph, we plot Kaplan-Meier survival curves for the cases predicted to have above average risk and the cases predicted to have below average risk. It is important to note that the risk group for each case was determined based on a predictor that did not use that case in any way in its construction. Hence, the Kaplan-Meier curves are essentially unbiased and the separation between the curves gives a fair representation of the value of the expression profiles for predicting survival risk.”
