# Supplementary material for: An 8-gene qRT-PCR-based gene expression score that has prognostic value in early breast cancer
Source: BMC Cancer. 2010 Jun 28;10:336. doi: 10.1186/1471-2407-10-336 (PMC2906483; doi:10.1186/1471-2407-10-336)
Supplement: Additional file 3 — Table S2, REMARK criteria. checking of REMARK criteria for marker development throughout text [file 1471-2407-10-336-S3.DOC]

Supplementary table 2. REMARK criteria used in this study.

| **Recommendation** | **Check** | **Place in manuscript** |
| --- | --- | --- |
| **Introduction** | | |
| 1. State marker, objectives, hypotheses | √ | Last paragraph |
| **Material & Methods** | | |
| 2. Patients, inclusion/exclusion criteria  3. Treatments received and how chosen  4. Type of biological material  5. Assay method and detailed protocol  6. Method of case selection  7. Endpoints  8. Variables initially examined  9. Sample size  10. Statistical methods, model building  11. Cutpoint determination | √  √  √  √  √  √  √  --  √  √ | 1^st^ paragraph  1^st^ paragraph  2^nd^ paragraph  2^nd^ and 3^rd^ paragraphs  1^st^ paragraph  “Methodology to find…”  Size, nodes, grade, genes  “Stat. analysis” & “Methodology to find…”  “Methodology to find...” |
| **Results** | | |
| 12. Flow of patients  13. Demographics  14. Relation of marker to other variables  15. Univariate analysis  16. Multivariate analysis  17. Confidence intervals  18, Further investigations | √  √  --  √  √  √  √ | 1^st^ paragraph  Table 1  Figure 3 & suppl. table 3  Table 3  Table 3  Other data sets, last parag |
| **Discussion** | | |
| 19. Interpret results, limitations  20. Future research and clinical value | √  √ | 7^th^ paragraph  Last paragraph |

Supplementary table 3. Univariate analysis with pathological factors.

|  | HR | 95.0% CI | | p |
| --- | --- | --- | --- | --- |
| Node | 3.223 | 1.611 | 6.448 | 0.001 |
| Grade  1vs3  2vs3 | 1.308  7.894 | 0.264  1.866 | 6.486  33.397 | 0.000  0.742  0.005 |
| Size | 3.599 | 1.629 | 7.953 | 0.002 |
