# Supplementary material for: An 8-gene qRT-PCR-based gene expression score that has prognostic value in early breast cancer
Source: BMC Cancer. 2010 Jun 28;10:336. doi: 10.1186/1471-2407-10-336 (PMC2906483; doi:10.1186/1471-2407-10-336)
Supplement: Additional file 4 — Table S3, univariate analysis. table displaying univariate analysis for distant metastasis-free survival and overall survival [file 1471-2407-10-336-S4.DOC]

Supplementary table 3. Univariate analysis with pathological factors.

|  | HR | 95.0% CI | | p |
| --- | --- | --- | --- | --- |
| Node | 3.223 | 1.611 | 6.448 | 0.001 |
| Grade  1vs3  2vs3 | 1.308  7.894 | 0.264  1.866 | 6.486  33.397 | 0.000  0.742  0.005 |
| Size | 3.599 | 1.629 | 7.953 | 0.002 |
