# Supplementary material for: An 8-gene qRT-PCR-based gene expression score that has prognostic value in early breast cancer
Source: BMC Cancer. 2010 Jun 28;10:336. doi: 10.1186/1471-2407-10-336 (PMC2906483; doi:10.1186/1471-2407-10-336)
Supplement: Additional file 5 — Table S4, DMFS in online databases. table displaying percentages of patients in low and high risk groups, as well as distant metastasis-free survival percentages in online available databases. [file 1471-2407-10-336-S5.DOC]

Supplementary table 4. Comparison of the 8-gene Score and other gene profiles using the NKI online database

| Profile | Group | % good vs poor | DMFS at 5 years |
| --- | --- | --- | --- |
| 8-gene Score | All patients  N-  N+  ER+ | 52 vs 48  53 vs 47  52 vs 48  65 vs 35 | 85.7 ± 2.7 vs 54.8 ± 4.3  86.1 ± 3.9 vs 50.5 ± 6.2  89.0 ± 3.7 vs 59.1 ± 6.1  88.8 ± 2.6 vs 54.2 ± 5.7 |
| 70-Gene Signature | All patients  N-  N+  ER+ | 39 vs 61  40 vs 60  38 vs 62  50 vs 50 | 94.7 ± 2.1 vs 60.5 ± 3.8  93.4 ± 3.2 vs 56.2 ± 5.5  95.2 ± 2.6 vs 66.3 ± 5.2  92.9 ± 2.4 vs 58.2 ± 4.7 |
| Recurrence Score | All patients | 35 vs 65 | 92.2 vs 58.5 |
| Wound Response | All patients | 23 vs 77 | 92.5 vs 63.6 |
